# MYC-repressed long noncoding RNAs antagonize MYC-induced cell proliferation and cell cycle progression

**DOI:** 10.18632/oncotarget.3909

**Published:** 2015-05-11

**Authors:** Taewan Kim, Ri Cui, Young-Jun Jeon, Paolo Fadda, Hansjuerg Alder, Carlo M. Croce

**Affiliations:** ^1^ Department of Molecular and Cellular Oncology, The University of Texas MD Anderson Cancer Center, Houston, TX, USA; ^2^ Department of Molecular Virology, Immunology and Medical Genetics, Comprehensive Cancer Center, The Ohio State University, Columbus, OH, USA

**Keywords:** MYC, long noncoding RNA, cell proliferation, cell cycle

## Abstract

The transcription factor MYC is a proto-oncogene regulating cell proliferation, cell cycle, apoptosis and metabolism. The recent identification of MYC-regulated long noncoding RNAs (lncRNAs) expands our knowledge of the role of lncRNAs in MYC functions. Here, we identify MYC-repressed lncRNAs named MYCLo-4, -5 and -6 by comparing 3 categories of lncRNAs (downregulated in highly MYC-expressing colorectal cancer, up-regulated by MYC knockdown in HCT116, upregulated by MYC knockdown in RKO). The MYC-repressed MYCLos are implicated in MYC-modulated cell proliferation through cell cycle regulation. By screening cell cycle-related genes regulated by MYC and the MYC-repressed MYCLos, we identified the MYC-repressed gene *GADD45A* as a target gene of the MYC-repressed MYCLos such as MYCLo-4 and MYCLo-6.

## INTRODUCTION

MYC is a transcription factor regulating global gene expression through heterodimerization with the protein myc-associated factor X (MAX) [1–3]. Through the genome-wide regulation of gene expression, MYC is involved in diverse cellular processes including cell growth, cell cycle, apoptosis, angiogenesis, cell differentiation and genomic instability [4]. MYC has also been found to be implicated in various biological phenomena and diseases such as healthy life span, Alzheimer's disease, Huntington disease, Parkinson disease and cancer [5–7].

The majority of MYC studies focus on its function in cancer due to the critical role of MYC in human cancer [5]. MYC is primarily overexpressed and/or amplified in numerous types of cancer through various mechanisms such as retroviral transduction and MYC translocation [4, 5, 8]. In addition, posttranscriptional regulation of MYC also contributes on the overexpression of MYC in cancer. For instance, the stability of MYC protein modulated by phosphorylation is regulated by promyelocytic leukemia zinc finger (*PLZF*) protein and long noncoding RNA PVT1 [9, 10].

Of the various members of noncoding RNAs, long noncoding RNAs (lncRNAs) are characterized by their size (larger than 200 nucleotides). Recently, many studies have revealed versatile functions of lncRNAs in gene expression modulation through various routes such as transcriptional, posttranscriptional, posttranslational and epigenetic regulation [10, 11]. Differential expression of the lncRNAs in various diseases implies the potential role of lncRNAs as therapeutic targets in the diseases [12]. Likewise, a cohort of lncRNAs has been reported and investigated in cancer. For instance, 8 CCAT family members from CCAT1 (CARLo-5) to CCAT8 were identified in colorectal cancer (CRC) [13–16]. Numerous PCAT family members were also found in prostate cancer [17]. In addition, a few lncRNAs such as BCAR4 and HOTAIR have a role in cancer metastasis [18, 19].

Recent studies profiled and identified lncRNAs that are regulated by MYC [13, 20, 21]. These studies show that many lncRNAs are commonly found to be regulated by MYC in various types of cancer. This suggests that MYC-regulated lncRNAs could have a critical role in MYC function. For example, MYCLo-1 and MYCLo-3 are regulated by MYC not only in B cell but also in various types of cancer, suggesting their critical role in MYC-driven cancer [13, 20]. Indeed, those MYC-regulated lncRNAs are involved in MYC-mediated cell cycle and tumorigenesis [13].

Here, we identify and report MYC-repressed lncRNAs that are also implicated in MYC-function by using the data of lncRNA microarray. To identify MYC-induced lncRNAs in our previous study, we compared 3 groups: lncRNAs upregulated in CRC cells and tissues with high MYC expression, lncRNAs downregulated by MYC knockdown in CRC cells such as HCT116 and RKO [13]. In this study, to identify MYC-repressed lncRNAs, we compared lncRNAs downregulated in CRC with high MYC expression, lncRNAs upregulated by MYC knockdown in the two different cell lines. As a result, we identified 3 lncRNAs named MYCLo-4, -5 and -6. We show that these MYC-repressed lncRNAs inhibit cell proliferation and that MYC induces cell proliferation by repressing these lncRNAs in cancer cells. Profiling of cell cycle-related genes regulated by the lncRNAs indicates that the MYC-repressed lncRNAs induce the expression of various cell cycle-related genes including *GADD45A* followed by cell cycle arrest. These results show the importance of MYC-repressed lncRNAs in the regulation of MYC downstream genes and MYC functions.

## RESULTS

### Identification of lncRNAs repressed by MYC

**Figure 1:**
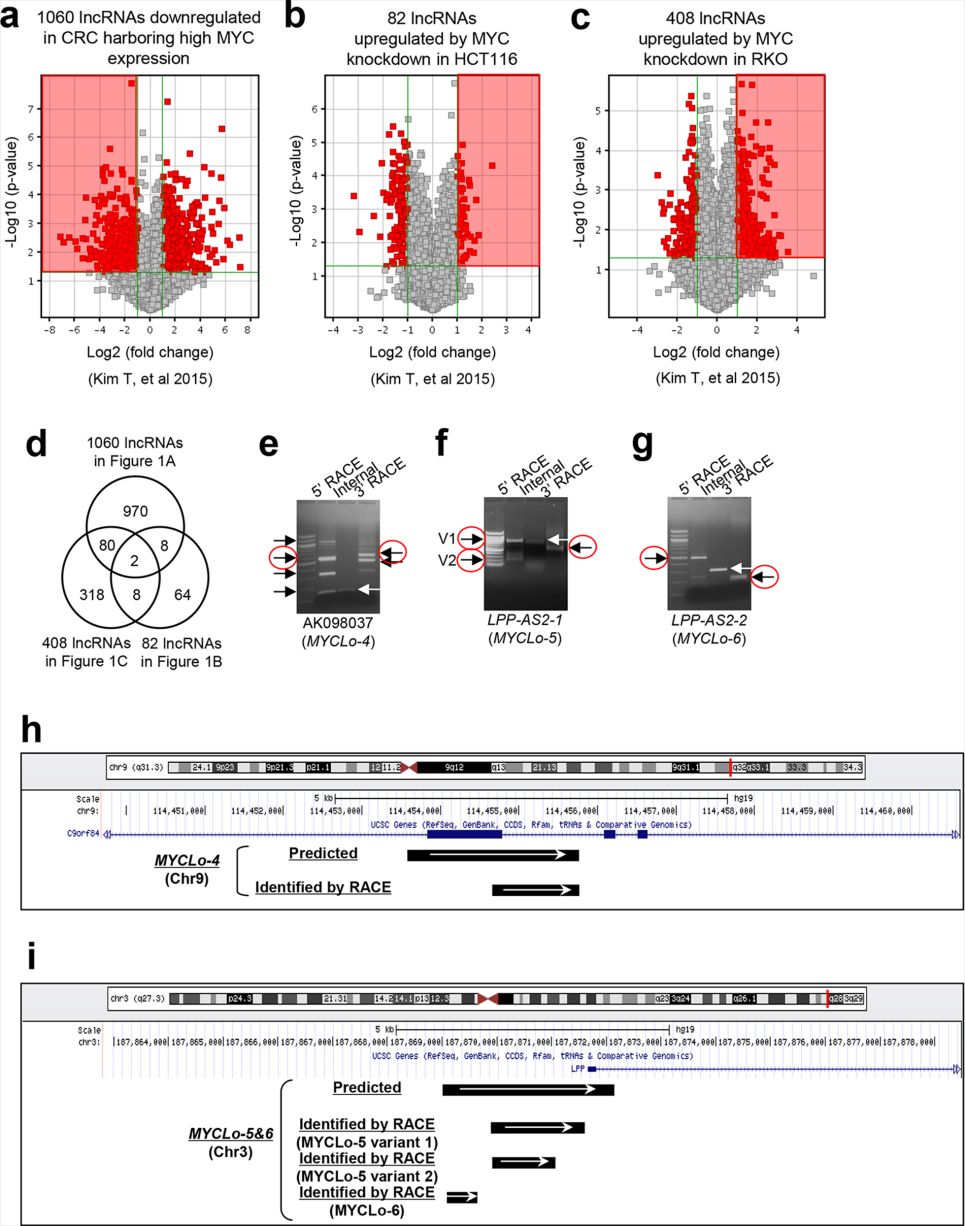
Identification of MYC-repressed lncRNAs **a.** Heatmap of lncRNAs dysregulated in CRC. LncRNAs downregulated in CRC with high MYC expression are highlighted with a red-colored box. **b.** and **c.** Heatmaps of lncRNAs dysregulated by *MYC* knockdown in HCT116 (b) or RKO (c). LncRNAs upregulated by MYC knockdown are highlighted with a red-colored box. **d.** Venn diagrams showing the number of downregulated lncRNAs in a *MYC*-dependent manner. **e–g.** 5′ (left lane) and 3′ (right lane) RACE and amplification of internal region (middle lane) covering the both starting points of 5′ and 3′ RACE. Black arrows indicate appropriate 5′ and 3′ RACE products and white arrows indicate PCR products of the internal region. The bands were confirmed by direct sequencing. The arrows circled in red indicate PCR products of the sequence deposited in GenBank. **h.** and **i.** Schematics of *MYCLo* structures and genomic localizations.

Recently, we identified lncRNAs dysregulated by MYC [13]. By profiling lncRNA expression in CRC, we found that about 7% of lncRNAs are dysregulated in CRC and that 1060 lncRNAs are downregulated in CRC (Figure 1a) [13]. Because MYC is overexpressed in the CRC cells and tissues used in the previous study, a part of the lncRNAs could be repressed by MYC. To further verify the repression of the lncRNAs by MYC, we also identified lncRNAs upregulated by MYC knockdown in HCT116 (Figure 1b) and RKO (Figure 1c) [13]. The comparison of the 3 categories shows 2 lncRNAs commonly found in the all 3 categories, namely AK098037 and LPP-AS2 (Figure 1d).

As shown in our previous lncRNA identification, predicted sequences of lncRNAs differ frequently with the actual sequences [13, 16]. Using rapid amplification of cDNA ends (RACE), we investigated the actual sequences of those lncRNAs (Figure 1e–1g). As shown, the 2 lncRNAs are shorter than the predicted sequences. In addition, we found that LPP-AS2 is expressed as 2 different lncRNAs (Figure 1f & 1g). Here we call these MYC-repressed lncRNAs, MYCLo-4 (AK098037, chromosome 9), MYCLo-5 (LPP-AS2–1, chromosome 3) and MYCLo-6 (LPP-AS2–2, chromosome 3) (Figure 1h & 1i). We also confirmed the expression of the lncRNAs by using Northern blot (Figure S1). Of the lncRNAs, MYCLo-5 is expressed as two variants, MYCLo-5 v1 and MYCLo-5 v2 (Figure 1i).

### Validation of MYC-mediated repression of MYCLo-4∼6 in various cancer types

**Figure 2:**
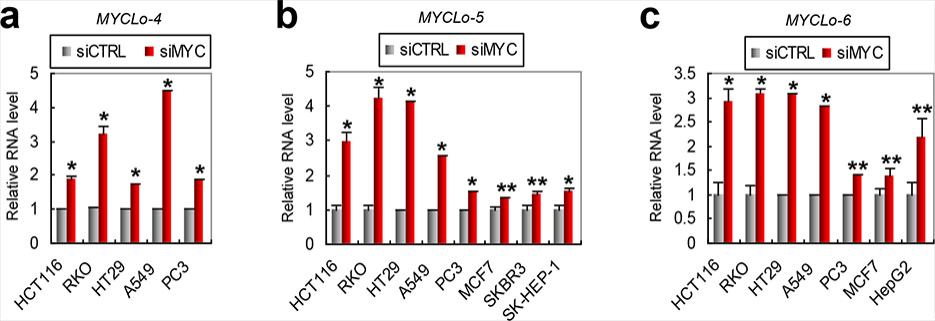
Confirmation of *MYC*-mediated regulations of MYC-repressed *MYCLos* in various cancers **a–c.** qRT PCR results showing MYC-mediated negative regulation of *MYCLo-4* (a), *-5* (b) and *-6* (c) in various types of cancer cells. Data are mean ± S.D. of three independent experiments and each measured in triplicate (**, *p* < 0.05; *, *p* = < 0.01).

MYC is frequently amplified and/or overexpressed in various types of cancer. By using Taqman assay, we tested the repression of MYCLo-4∼6 by MYC in various cancer types. Of the tested cell lines, MYCLo-4 was upregulated by MYC knockdown in HCT116 (CRC), RKO (CRC), HT29 (colorectal adenocarcinoma), A549 (Lung carcinoma) and PC3 (prostate cancer) (Figure 2a). The other 2 lncRNAs, MYCLo-5 and -6 were also induced by MYC repression in various cancer types such as CRC (HCT116, RKO and HT29), lung cancer (A549), prostate cancer (PC3), breast cancer (MCF7 and SKBR3) and hepatocellular carcinoma (SK-HEP-1 and HepG2) (Figure 2b & 2c). These results indicate that MYCLo-4∼6 are common targets of MYC not only in CRC but also in other types of cancer.

### Identification and characterization of promoters of MYCLo-4∼6

**Figure 3:**
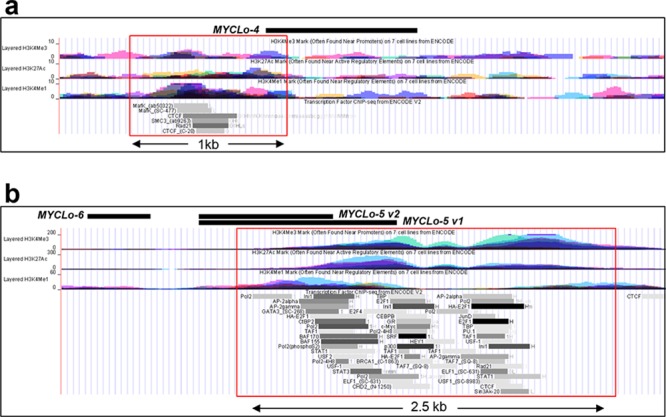
Identification and characterization of promoters of MYC-repressed MYCLos **a.** and **b.** ENCODE database showing promoter and regulatory activity markers such as H3K4Me1, H3K4Me3 and H3K27Ac, and ChIP-seq database showing transcription factors that bind to the promoters of MYC-repressed MYCLos.

MYC is a transcription factor that directly interacts with target gene promoters, leading to the enhancement of target gene expression [2, 3]. However, large number of MYC-targeted genes and noncoding RNAs are also repressed by direct or indirect targeting by MYC [2, 3, 13]. To scrutinize the regulatory mechanism of MYCL-4∼6 by MYC, we analyzed the ENCODE database [22]. Using the database of H3KMe1 mark, H3KMe3 mark and H3K27Ac mark, we identified 5′ upstream regions of MYCLo-4∼6 that show promoter and regulatory activities (Figure 3a & 3b). The ENCODE transcription factor ChIP-seq database also shows that various transcription factors interact with the promoter regions of MYCLo-4∼6. In the promoter of MYCLo-4, MYC is not shown to bind, supporting indirect repression of MYCLo-4 transcription by MYC (Figure 3a). In the database, several transcription factors such as MAFK, CTCF, SMC3 and RAD21 are shown to interact with the promoter of MYCLo-4. On the other hand, it is shown that MYC binds to the promoter of MYCLo-5 and -6, suggesting direct regulation of MYCLo-5/6 by MYC. However, MYC binding is only seen in a few cell lines in the database although we show MYC-mediated repression of MYCLo-5/6 in various cancer cell lines (Figure 2a–2c). These also indicate the possibility of indirect repression of MYCL-5∼6 by MYC. Indeed, numerous transcription factors interact with the promoter of MYCL-5/6 in the database (Figure 3b).

### MYC-repressed MYCLos prohibit MYC-enhanced cell proliferation through cell cycle regulation

**Figure 4:**
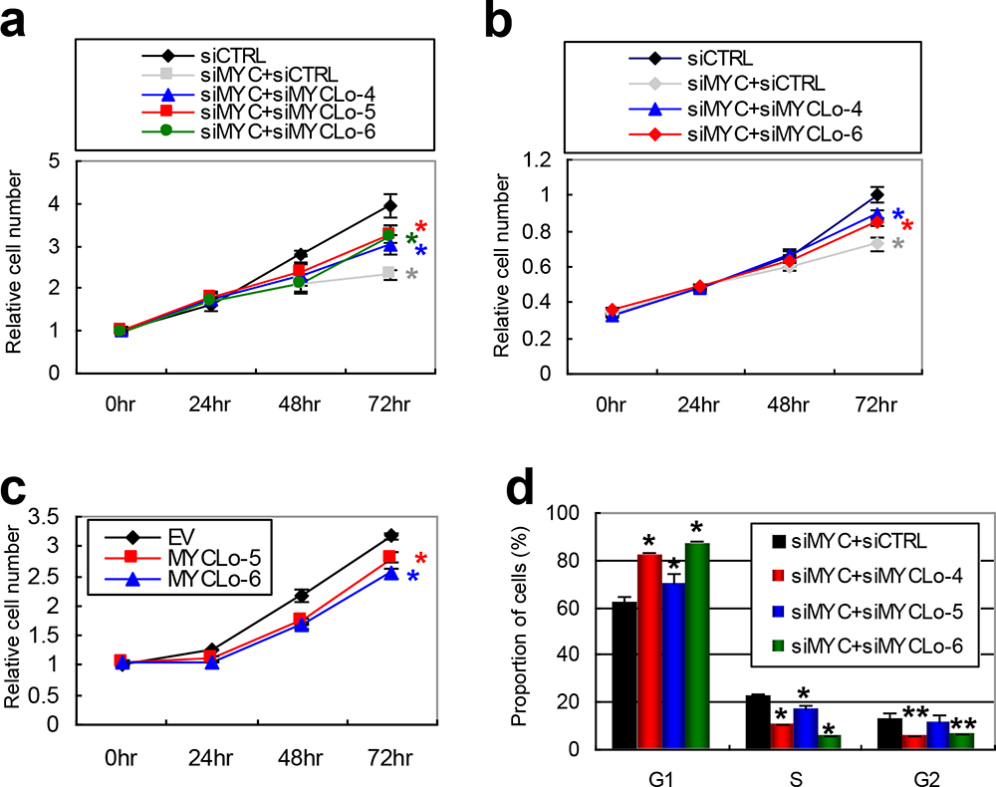
MYC-repressed MYCLos inhibit cell proliferation through cell cycle regulation in a MYC-dependent manner **a.** and **b.** Cell proliferation assays. PC3 (a, *n* = 4), RKO (b, *n* = 4) cells were treated with siRNAs (25nM+25nM) as indicated and subjected to proliferation assay every 24 hr. Data are mean ± S.D. of four independent experiments (*, *p* = < 0.01). **c.** Cell proliferation assay. HCT116 cells were transfected with pcDNA3.3 plasmids expressing *MYCLo-5* or *-6* and subjected to a cell proliferation assay every 24 hr (*n* = 3). Data are mean ± S.D. of three independent experiments (*, *p* = < 0.01). **d.** Relative cell number in each cell-cycle phase determined by flow cytometry analysis. PC3 cells were treated with siRNAs (25nM+25nM) as indicated and analyzed 48 hr after transfection. Data are mean ± S.D. of three independent experiments (**, *p* < 0.05; *, *p* = < 0.01).

To investigate the function of MYC-repressed MYCLos, we examined the effect of knockdown of the MYCLos in cell proliferation. The knockdown of MYC by MYC siRNA decreased cell proliferation, however, simultaneous knockdown of MYC and MYC-repressed MYCLos partially rescues the inhibition of cell proliferation caused by MYC knockdown in PC3 and RKO cells (Figure 4a & 4b). In addition, exogenous expression of MYC-repressed MYCLos also inhibited cell proliferation in HCT116 cells (Figure 4c, Figure S2). These results indicate that the induction of MYC-repressed MYCLos led by MYC knockdown is implicated in decreased cell proliferation caused by MYC knockdown.

MYC induces cancer cell proliferation through cell cycle regulation [4]. Likewise, knockdown of the MYC-repressed MYCLos alters the cell cycle. Flow cytometry analysis shows that repression of MYCLo-4 or -6 induced by MYC knockdown suppresses G2 arrest and that inhibition of MYCLo-5 induced by MYC knockdown increases the cell population in S phase (Figure 4d). These results suggest that MYC involves the MYC-repressed MYCLos in cell cycle regulation.

### MYC-repressed lncRNAs participate in regulation of MYC target genes including GADD45A

**Figure 5:**
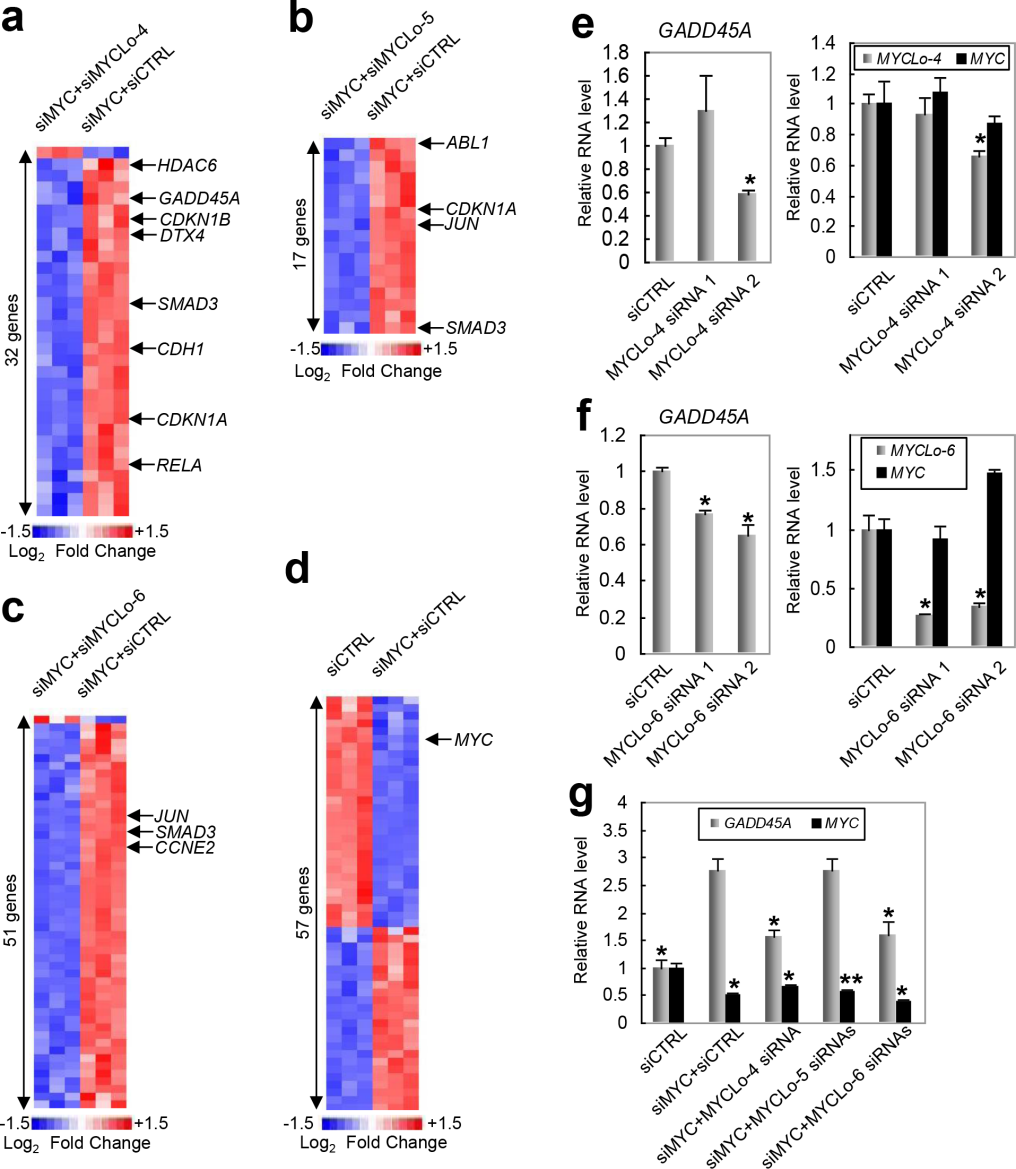
Profiling of cell cycle-related genes regulated by MYC-repressed MYCLos **a–c.** Heatmaps representing cell cycle-related genes dysregulated by MYCLo-4 (a), -5 (b) and -6 (c) in PC3 cells. The genes that are oppositely regulated by MYC and MYC-repressed lncRNAs are indicated. Expression values displayed in gradient of red and blue are Log_2_-transformed fold change. The list of the dysregulated genes by MYC knockdown is available in Tables S1 (a), S2 (b) and S3 (c). **d.** Heatmaps representing cell cycle-related genes dysregulated by MYC in PC3 cells. MYC downregulation by siMYC is indicated. Expression values displayed in gradient of red and blue are Log2-transformed fold change. The list of the dysregulated genes by MYC knockdown is available in Table S4. **e.** and **f.** qRT-PCR results showing MYC-independent regulation of GADD45A by MYCLo-4 (e) or -6 (f) knockdown. CCD-18Co cells were treated with 50nM siRNAs targeting MYCLo-4 for 72 hr. Data are mean ± S.D. of three independent experiments and each measured in triplicate (**, *p* < 0.05; *, *p* = < 0.01). **g.** qRT-PCR results showing that *MYC*-repressed *GADD45A* expression is dependent on *MYCLo-4* and *-6*. PC3 cells were treated with the indicated siRNAs (50 nM) for 72 hr. Data are mean ± S.D. of three independent experiments and each measured in triplicate (**, *p* < 0.05; *, *p* = < 0.01).

To identify the cell cycle regulators modulated by the MYC-repressed MYCLo-4, -5 and -6, we profiled the expression of 183 cell cycle regulator genes using the nCounter Virtual Cell Cycle Gene Set from NanoString Technologies (Materials and Methods). We identified 32 genes, 17 genes and 52 genes significantly dysregulated by knockdown of MYCL-4, -5 or -6, respectively (more than 1.5 fold change, *p*-value < 0.05) (Figure 5a–5c, Table S1–S3). Most of the dysregulated genes among the 183 cell cycle regulators are repressed by knockdown of the MYC-repressed MYCLos, suggesting that MYC-repressed lncRNAs modulate the cell cycle by enhancing the expression of cell cycle-related genes. By comparing the genes regulated by MYC (Figure 5d, Table S4), we further identified subsets of genes commonly regulated by MYC and the MYCLos under our selection criteria (more than 1.5 fold change, *p*-value < 0.05), as indicated in Figure 5a–5c.

The critical regulator of G2 arrest, *GADD45A* is known to be repressed by MYC expression [23]. Interestingly, the results of the NanoString Gene Expression Assay show that the knockdown of MYCLo-4 suppresses *GADD45A* expression (Figure 5a). Although GADD45A is not indicated in Figure 5c due to our selection criteria (more than 1.5 fold change, *p*-value < 0.05), data analysis shows that knockdown of MYCLo-6 also induces GADD45A expression (about 1.4 fold change, *p*-value < 0.05). We verified by qRT-PCR the reduction of the *GADD45A* mRNA level by MYCLo-4 knockdown using MYCLo-4 siRNA-2 (Figure 5e). On the other hand, nonfunctional MYCLo-4 siRNA-1 did not repress the *GADD45A* expression level. Additionally, we verified the repression of *GADD45A* expression by knockdown of MYCLo-6 using qRT-PCR (Figure 5f). Consistent with the data of the NanoString Gene Expression Assay, however, MYCLo-5 knockdown showed no inhibitory effect in *GADD45A* expression (Figure S3). These results show the role of the MYCLo-4 and -6 in MYC-mediated *GADD45A* suppression. To further verify the involvement of the MYCLos in the MYC-mediated repression of *GADD45A* expression, we examined the expression of *GADD45A* altered by single knockdown of MYC or simultaneous knockdown of MYC and each MYCLo-4/6. The *GADD45A* expression induced by MYC knockdown is significantly repressed by knockdown of MYCLo-4 or -6 (Figure 5g, Figure S4). The results strongly support that MYCLo-4 and -6 are important mediators in MYC-mediated *GADD45A* repression.

## DISCUSSION

In addition to MYC-induced lncRNAs such as MYCLo-1∼3, we identified different MYCLo family members, MYC-repressed MYCLo-4∼6. The proto-oncogene MYC, frequently overexpressed in various cancers, has a key role in tumor cell cycle regulation and tumor pathogenesis [4, 5, 8]. As we recently showed, the MYC-induced MYCLos are involved in MYC functions including cell cycle regulation and tumorigenesis. Likewise, the MYC-repressed MYCLos are also implicated in MYC-mediated cell proliferation and cell cycle regulation. And MYC-repressed MYCLos as well as MYC-induced MYCLos are also regulated by MYC in various cancer types [13, 20, 21]. Taken together, these results emphasize the critical role of MYC-regulated lncRNAs in MYC function and MYC-mediated regulation of the MYCLos in wide range of cancer types.

**Figure 6:**
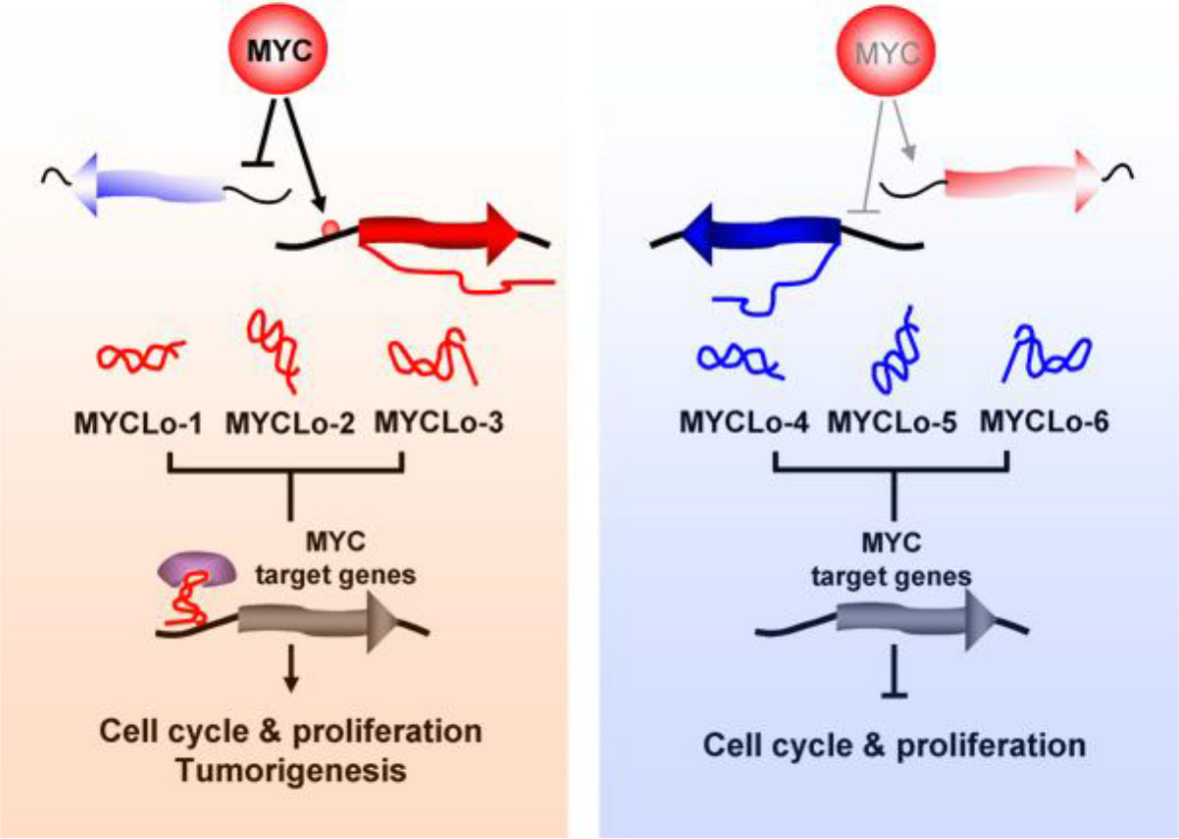
Functional mechanisms of MYC-regulated lncRNAs in MYC functions

More than one third of MYC target genes are repressed by MYC although the transcription factor MYC is a universal amplifier of global gene expression [2, 3]. Many studies have shown the mechanisms of MYC-mediated repression of target genes through the interaction with initiator element, C/EBP, AP2 or Miz-1 [24–28]. And findings of MYC-regulated microRNAs enabled to elucidate an additional mechanism of MYC-mediated target gene repression through small noncoding RNAs [29–31]. A recent study also implicated long noncoding RNAs (lncRNAs) in the mechanism of repression of MYC target genes, showing that MYC-induced lncRNAs suppress MYC-repressed target genes [13]. Here, we show that MYC-repressed lncRNAs also regulate MYC-repressed target genes by enhancing their expression (Figure 6). This finding provides an extension of our understanding of MYC-regulated lncRNAs in MYC function.

Recently we showed that MYC-induced lncRNAs such as MYCLo-1 and -2 regulate the transcription of known MYC-repressed target genes such as CDKN1A (p21) and CDKN2B (p15) [13]. The two genes produce critical cell cycle regulators in G1 arrest [32, 33]. Another MYC-repressed target, GADD45A is known as a regulator of G2 cell cycle arrest [34]. Here, we show that MYC-repressed lncRNAs such as MYCLo-4 and -6 are involved in the regulation of *GADD45A*, the MYC-repressed target gene. The MYC-induced MYCLos repress the expression of CDKN1A and CDKN2B. On the contrary, the MYC-repressed MYCLos regulates the MYC-repressed target gene, GADD45A by inducing the expression. Furthermore, our results from cell cycle-related genes profiling show that most genes regulated by the MYC-repressed MYCLos are repressed by inhibition of the MYCLos suggesting the enhancer-like function of the MYC-repressed MYCLos. Our results show a different mode of lncRNA-mediated MYC function. Further studies are required in order to understand the MYC-mediated regulatory mechanisms of MYC-repressed MYCLos and the role of the MYCLos in MYC function.

Some transcription factors such as MYC and p53 have a critical role in cancer development by regulating transcription of numerous genes. For the last decade, it has been revealed that those transcription factors are responsible for the regulation of not only protein coding genes but also noncoding RNAs, and the importance of the noncoding RNAs has been elucidated [29, 31, 35–37]. MYC-regulated lncRNAs were recently highlighted, compared to other noncoding RNAs regulated by cancer-associated transcription factors [13, 20, 21]. In this study, we identify MYC-repressed lncRNAs and characterize their function in the MYC pathway. Together with the previous findings of MYC-regulated lncRNAs, our results show that MYC tunes its oncogenic function by inducing oncogenic lncRNAs and repressing tumor-suppressor lncRNAs (Figure 6).

## MATERIALS AND METHODS

### NanoString nCounter gene expression assay and data analysis

NanoString Technologies' nCounter Virtual Cell Cycle Gene Set was used following manufacturer's instructions (NanoString Technologies). Briefly, total RNA (100ng) was used as input for nCounter mRNA sample preparation reactions. All sample preparation was performed according to manufacturer's instructions (NanoString Technologies). Hybridization reactions were performed according to manufacturer's instructions with 5 μl of the 5-fold diluted sample preparation reaction. All hybridization reactions were incubated at 65°C for a minimum of 16 hrs. Hybridized probes were purified and counted on the nCounter Prep Station and Digital Analyzer (NanoString Technologies) following the manufacturer's instructions. For each assay, a high-density scan (600 fields of view) was performed. Data analysis was performed using the nSolver analysis software (NanoString Technologies) and dChip software.

### Rapid amplification of cDNA ends (RACE)

5′ and 3′ RACE were performed using the SMARTer RACE cDNA Amplification Kit (Clontech). All procedures were done in accordance with manufacturer's instruction. Total RNA from HT29 or SW620 was used. PCR of the internal region was performed when starting points of 5′ and 3′ RACE had an unamplified gap. All primers used for RACE are presented in Table S5.

### Cells, oligonucleotides and transfection

All cell lines were cultured as recommended by the ATCC. All custom siRNAs were designed by the Dharmacon custom siRNA design tool based on sequence information identified by RACE. For each MYCLo, 2 kinds of custom siRNAs were designed and used. All sequence information of the siRNAs is shown in Table S6. Cells were transfected with Lipofectamine RNAiMAx (Invitrogen) for oligonucleotides and Lipofectamine LTX for plasmids in accordance with manufacturer's instructions. In all transfection experiments, 50nM siRNA was used.

### Quantitative real-time PCR (qRT-PCR)

Total RNA was prepared from cells using TRIZOL (Invitrogen) in accordance with manufacturer's instructions. Total RNA was subjected to quantitative real-time PCR (qRT-PCR). RNA levels were analyzed using TaqMan Gene Expression Assays, in accordance with manufacturer's instructions (Applied Biosystems). All RT reactions, including no-template controls and RT minus controls, were run in a GeneAmp PCR 9700 Thermocycler (Applied Biosystems). RNA concentrations were determined with a NanoDrop 2000 Spectrophotometer (ThermoFisher Scientific). Samples were normalized to GAPDH or OAZ1 for mRNAs and lncRNAs (Applied Biosystems). Gene expression levels were quantified using the 7900 HT Fast Realt-Time PCR System (Applied Biosystems). Comparative real-time PCR was performed in triplicate, including no-template controls. Relative expression was calculated using the comparative Ct method. Probes used for Taqman Assays are shown in Table S6.

### Cell proliferation assay

For cell proliferation assay, the MTS assay from Promega (CellTiter 96 AQueous One Solution Cell Proliferation Assay) was used following manufacturer's instruction. Briefly, cells in 96-wells plate were incubated for 72 hr in a humidified 5% CO_2_ atmosphere after transfection with indicated siRNAs, followed by addition of 20μl CellTiter 96 AQueous One Solution and 1–4 hr incubation in humidified 5% CO_2_ atmosphere. The absorbance at 490nm was recorded.

### Flow cytometry analysis

For DNA content analysis, cells were fixed in methanol at −20° C, washed again, rehydrated, re-suspended in PBS containing 2 μg/ml propidium iodide (PI) and 5 μg/ml RNase A, and analyzed by BD FACS Calibur Flow Cytometer.

### Statistics

All graph values represent means ± S.D. from three independent experiments with each measured in triplicate. The differences between two groups were analyzed with unpaired two-tailed Student's *t*-test. *p*-value < 0.05 was considered statistically significant and indicated with asterisks as described in figure legends.

## SUPPLEMENTAY FIGURES AND TABLES














