# Supplementary material for: MYC-repressed long noncoding RNAs antagonize MYC-induced cell proliferation and cell cycle progression
Source: Oncotarget. 2015 May 11;6(22):18780–9. doi: 10.18632/oncotarget.3909 (PMC4662455; doi:10.18632/oncotarget.3909)
Supplement: Supplementary file 1 [file oncotarget-06-18780-s001.pdf]

SUPPLEMENTAY FIGURES AND TABLES

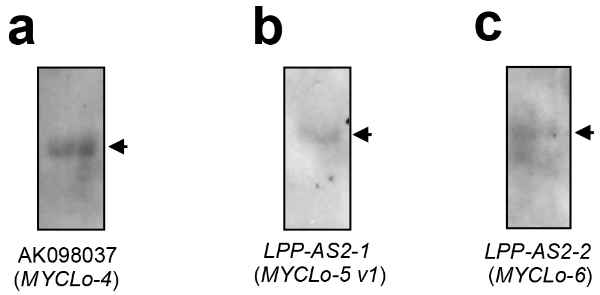

Supplementary Figure S1 (related to figure 1): Confirmation of expression of MYC-repressed lncRNAs. Verification of MYCLo-4, -5 and -6 expression with Northern blot anaysis.

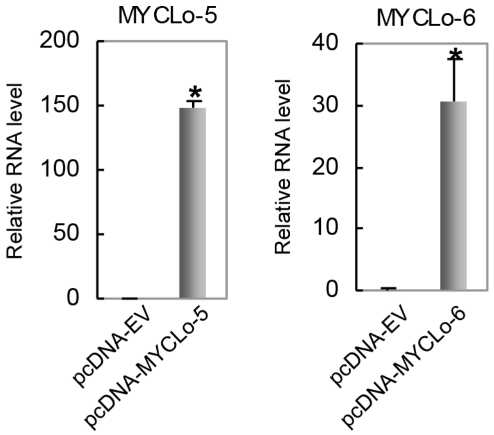

Supplementary Figure S2 (related to figure 4): Confirmation of exogenous expression of MYCLo-5 & -6. Verification of MYCLo-5 and -6 expression levels in HCT116 cells transfected with pcDNA3.3 control vector (EV) or pcDNA3.3-MYCLo-5/6 vectors. Data are mean ± S.D. of three independent experiments and each measured in triplicate (*, p = < 0.01).

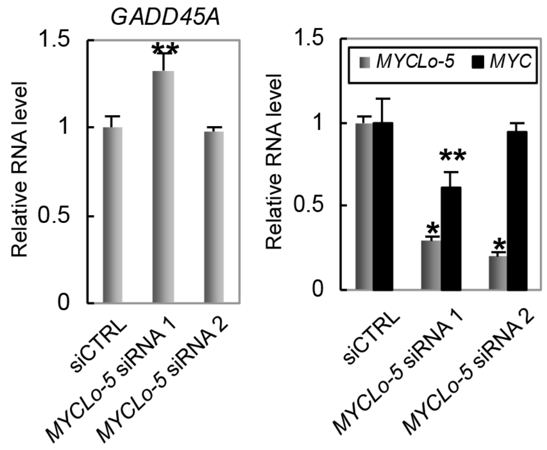

Supplementary Figure S3 (related to figure 5): No effect of MYCLo-5 knockdown on GADD45A expression. MYCLo-5 is not involved in regulation of GADD45A expression. qRT-PCR results showing that knockdown of MYCLo-5 does not affect GADD45A expression. Data are mean ± S.D. of three independent experiments and each measured in triplicate (**, $p = < 0.05$; *, $p = < 0.01$).

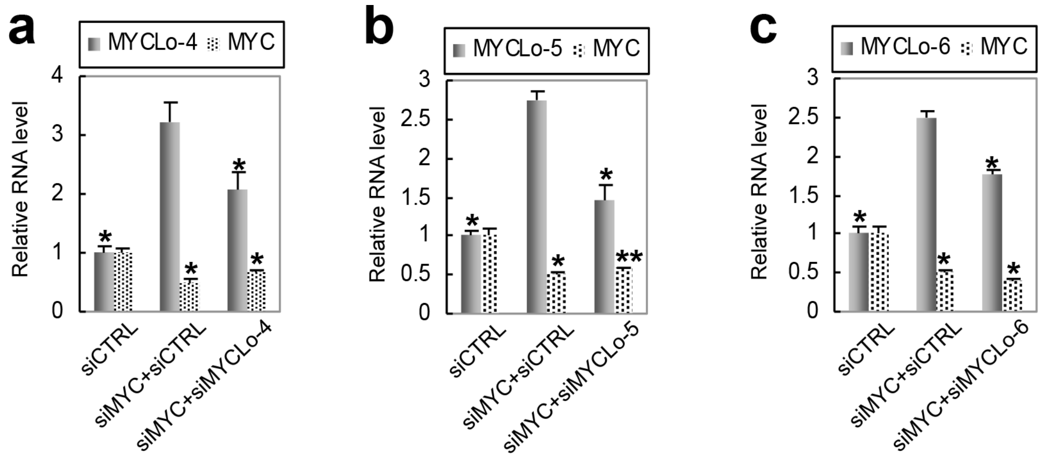

Supplementary Figure S4 (related to figure 5): Confirmation of MYC and MYCLos (MYCLo-4, -5 and -6) expression levels using qRT-PCR. Total RNA samples used in Fig. 5g were used to determine the expression levels of MYC **a–c.**, MYCLo-4 (a), MYCLo-5 (b) and MYCLo-6 (c). Data are mean ± S.D. of three independent experiments and each measured in triplicate (**, $p = < 0.05$; *, $p = < 0.01$).

Supplementary Table S1: The list of genes dysregulated by siMYCLo-4 in PC3.

Supplementary Table S2: The list of genes dysregulated by siMYCLo-5 in PC3.

Supplementary Table S3: The list of genes dysregulated by siMYCLo-6 in PC3.

Supplementary Table S4: The list of genes dysregulated by siMYC in PC3.

Supplementary Table S5: List of primers used for RACE, PCR, Sequencing and Cloning.

Supplementary Table S6: Information of custom and prebuilt siRNAs.
